# A design space for automated material handling vehicles

**DOI:** 10.3389/frobt.2023.1276258

**Published:** 2023-12-14

**Authors:** Alexander G. Mirnig, Peter Fröhlich, Setareh Zafari, Michael Gafert, Lukas Kröninger, Manfred Tscheligi

**Affiliations:** ^1^ Austrian Institute of Technology, Vienna, Austria; ^2^ Artificial Intelligence and Human Interfaces, University of Salzburg, Salzburg, Austria

**Keywords:** vehicle automation, material handling, interaction design, design space, human in the loop

## Abstract

Material Handling Vehicles (loaders, excavators, forklifts, harvesters, *etc.*) have seen a strong increase in automation efforts in recent years. The contexts such vehicles operate in are frequently complex and due to the often very specific nature of industrial material handling scenarios, know-how is fragmented and literature is not as numerous as, for example, for passenger vehicle automation. In this paper, we present a contextual design space for automated material handling vehicles (AMHV), that is intended to inform context analysis and design activities across a wide spectrum of material handling use cases. It was developed on the basis of existing context and design spaces for vehicle and machine automation and extended via expert knowledge. The design space consists of separate context and interaction subspaces, that separately capture the situation and each individual point of interaction, respectively. Implications, opportunities, and limitations for the investigation and design of AMHV are discussed.

## 1 Introduction

Along with the continuous automation of public and private transport as well as manufacturing environments, material handling is another context that sees increasing automation efforts (Machado et al., 2021a; Machado et al., 2021b; Li et al., 2018; Efthymiou and Ponis, 2019). Not only does the number of employed front loaders, excavators, bulldozers, forwarders, mobile cranes, and other material handling vehicles increase, but their degrees of automation do as well (Ha et al., 2018; Heath, 2018; Frank, 2019), thus increasing in-context complexity on two levels. Even without automation factored in, material handling is a complex context by itself: Not only does it involve navigation from one point to another, but destinations are usually also changing as tasks progress or are finished (e.g., moving from stack to stack as they are gradually filled at the same location or transitioning from one location to another entirely). On top of that, there is the non-navigational handling operation, e.g., grabbing, dredging, lifting, *etc.*, all mediated through higher degrees of freedom (e.g., cranes with multiple junction points) and resulting complex controls. In addition, material handling is needed in a wide variety of environments, many of which are not regular on-road environments (e.g., construction sites, farmland, gravel pits, forests, *etc.*). By adding automation to this already demanding mix, the additional challenge of adequately keeping the *human in the loop* (Gil et al., 2019) receives greater relevance.

It is unlikely that any given material handling situation is limited to a single handling operation of quantity X of material Y to a point Z. Rather, material handling exists along a process chain, often at multiple points, and in interaction with other agents, which can and often are themselves material handlers (e.g., loading containers via crane onto a train, then unloading container contents via forklift for a very common example with already three vehicle handlers involved). Depending on the levels of individual automation of each handler as well as the automation of the entire handling chain, properly calibrating the human-in-the-loop is not trivial: At which points in the handling flow does a human need to observe/verify/intervene? Which capacity/qualification does the human need to have, mediated by the task that needs to be performed? At which physical point does the interaction happen and does it need to be done on-site or can it be done remotely? Can the interaction be prompted by a system or must it be human-initiated? These and similar questions need to be answerable in order to properly and safely operate heavy machinery within a material handling context (Heath, 2018).

In Human-Computer Interaction (HCI), one of the best ways to properly capture a context is via a *design space*. A design space essentially is a “space of possibilities”, which organizes design opportunities and constraints along specified dimensions (Heape, 2007; Beaudouin-Lafon and Mackay, 2009; Biskjaer et al., 2014; MacLean et al., 2020). A comprehensive design space thus should capture and structure a given interaction context, including related stakeholders, points of interaction, and any variables that can influence the interaction between stakeholders and machines or devices within the context. The goal and purpose of a design space is then to show where within the space activities can be done, whom they will likely affect, and conversely what they are mediated by. This greatly aids interaction designers in planning where, when, and for whom to design - an essential step before the actual interaction design begins.

Currently, there is no such design space for automated material-handling vehicles. There are numerous related design spaces, including in-car interaction (Kern and Schmidt, 2009; Haeuslschmid et al., 2016; Wiegand et al., 2019) and external communication of automated vehicles (Colley et al., 2017; Colley and Rukzio, 2020). The transferability of these design spaces to material handling is limited, as material handling involves specific task types and interaction chains, driving maneuvers, and handling actions combined, as well as greater contextual variability due to the great variety of material handling scenarios. Due to the industrial nature of material handling use cases, there is quite a good number of automation projects with significant funding behind them, yet there is also little knowledge exchange between these projects, which would enable a common material handling automation knowledge base.

Thus, a design space for material handling would be both desirable and beneficial to 1) capture and categorize current efforts and 2) structure, guide, and help align future design and development efforts. In this paper, we present such a design space that was derived from components of related design spaces and enriched with aspects specific to material handling. The multidimensional design space structures the automated vehicle handling space via the dimensions *task and purpose*, *automation setting*, *situation*, and *interaction*. The design space allows specification of driving and handling tasks, mapping them to individual interaction points, and defining the role of the human not only in relation to the interactive device but also via levels of autonomy of (a) the vehicle, (b) the material handler, and (c) the operative process.

## 2 Related work

Heavy material handling vehicles are primarily or exclusively used on private, off-highway grounds - be it construction and mining grounds, industrial production sites, agricultural fields, or logistics areas. The higher control over processes, traffic, and lower regulatory demands have made material handling vehicles pioneers for automated transport. Automated Guided Vehicles (AGV) have been used for decades in specific industrial contexts (Wankhede and Vinodh, 2021), and these are increasingly used in one-to-many relationships through the remote management of driverless vehicle fleets (Fottner et al., 2021). There is a high business interest and considerable growth prospects with regard to achieving higher autonomy levels (Krug et al., 2019; Gupta et al., 2022). The strive for automatizing material handling vehicles is also motivated by ongoing driver shortage (Costello and Suarez, 2015), the need to increase the attractiveness of work within harsh environments, as well as to reduce safety risks (Machado et al., 2021b). However, despite the longstanding experience and growing relevance of such systems for automated handling of heavy materials, there is surprisingly little open scientific literature available about contextual factors and HMI design, and if available, it is scattered across different sub-disciplines (Krug et al., 2019; Machado et al., 2021b). For such situations with little knowledge about the context variables, general scope, and design alternatives, a design space can help to provide a generic means of orientation. In the following, the state of the art of design spaces is summarised. Then, taxonomies for describing the level of automation and contextual factors are described.

### 2.1 Design spaces in HCI

Design spaces have been used in architecture, computer science, and especially in Human-Computer Interaction as a complement to standards and guidelines, to inspire design decisions and innovations (Simon, 1975; Card and Mackinlay, 1997; Shaw, 2012; Haeuslschmid et al., 2016; Halskov et al., 2021). Their primary use is to structure and group designs and parameters according to a set of design dimensions. Each design option is ideally represented as a point within that space, thus defining the parameters for each of its constituting dimensions (Simon, 1975). While early work focused on fundamental classifications of input devices (Buxton, 1983; Card et al., 1991) and information visualization (Card and Mackinlay, 1997; Chi, 2000), important contributions have also been provided for specific types of interaction, such as mobile phone input (Ballagas et al., 2008), public displays or multimodal interaction (Müller et al., 2010). Since Kern’s and Schmidt’s design space for the car cockpit (Kern and Schmidt, 2009), further more specific automotive user interface aspects were addressed, such as augmented reality (Tönnis et al., 2009; Haeuslschmid et al., 2016; Wiegand et al., 2019), conversational interaction (Braun et al., 2017), multimodal interaction (Wang et al., 2022) as well as application contexts like the mobile office (Li et al., 2020). With regard to design support of automated driving, however, design spaces for the internal design of automated vehicles are still rare, but for the external communication of automated vehicles (Colley and Rukzio, 2020) and teleoperation, first proposals have been made (Graf et al., 2020). While surveys on interaction issues with AMHV have been put forward (Hoffmann and Chan, 2018), no design space is available to support the development of human-automation interaction for this category of systems.

### 2.2 Level of automation

With the constant penetration of automation and robotics in industrial contexts, the nature of human tasks and involvement with technology is changing (Chen and Barnes, 2014; Gil et al., 2019). The increasing intelligence and sophistication of systems enables human operators of AMHV to not only manually operate them (“in-the-loop”), but also to transition into a supervisory role (“on-the-loop”), where fleets of vehicles are monitored over a distance [see related definitions in Merat et al. (2019)] Various models within and across application areas to categorize the degree of automation and human involvement therein have been proposed [see Vagia et al. (2016), for a comprehensive overview). While for automation of passenger cars, the SAEJ3016 taxonomy of automation levels (Taxonomy, 2021) has become a *de facto* standard (despite other existing standards (Hopkins and Schwanen, 2021)], automation taxonomies for heavy machinery or load handling vehicles are mostly specific to application fields, such as agriculture (Benos et al., 2020), constructions sites (Lee et al., 2022), or mining (Rogers et al., 2019). Only recently, Machado et al. (Machado et al., 2021a) proposed an approach that makes reference to several preliminary models (Heath, 2018; Heikkilä et al., 2019; Krug et al., 2019), which is essentially constituted of a 2-dimensional matrix, where both for driving and for handling (or “manipulation”) the six levels of the SAEJ3016 are applied.

### 2.3 Contextual factors

Interaction design choices for AMHV will have to take account of various contextual factors, in order to achieve optimal system control and perception, worksite communication, and decision making. Only a few scientific accounts, notably all of them from the research area of Automotive UI, include contextual factors like the traffic situations and involved traffic participants, thus actually extending towards contextual design spaces (Wiegand et al., 2019; Colley and Rukzio, 2020; Graf et al., 2020; Colley et al., 2022). Taxonomies of context have a long tradition, as documented in the standard definition of “context of use” in ISO 9241-210 and ISO 20282-1 (for Standardization, 2010; ISO, 2006; Bevan et al., 2015) and 20 years of discussion on context-aware computing (Schmidt et al., 1999; Bradley and Dunlop, 2005; Bauer and Novotny, 2017; Dey, 2018). However, there is no dedicated taxonomy of physical, social, or organizational context factors for material handling vehicles, let alone related to their automation.

## 3 Methods

While there is no standard method for creating design spaces, we used a systematic procedure for developing the design space that consisted of a literature review as well as two design and evaluation cycles. The purpose of the initial literature review was to identify existing relevant design spaces to use as a basis. We used two iterative cycles so that we could do one in-depth evaluation and fundamental iteration and then a second refinement afterward, following a standard iterative approach. For practical relevance, we focused on the AMHV domains of construction, agriculture, intralogistics, and manufacturing, which are frequent subjects of automation efforts.

After defining the scope, we conducted the initial literature review across the ACM Digital Library and IEEE Xplore. These two data sources were chosen for literature review work since both ACM Digital Library and IEEE Xplore feature a wide selection of reliable HCI works. We used the following search queries in August 2022 in English-language publications: “automated/automation material handling vehicle”, “automated/automation crane”, “automated/automation forklift”. This resulted in a total of 908 publications (633 publications in ACM Digital Library and 275 publications in IEEE Xplore). After having the database, we screened the papers that met our criteria. First, we looked for papers that potentially had an example of design space by searching through their title, authors’ keywords, abstract, and introduction with the keywords “design space”. Second, as we found no single design space paper for any automated material handling vehicle, we instead focused on publications dealing with the automated vehicles. Third, we focused on detailed descriptions or full overviews of design spaces for the analysis and, therefore, targeted full conference or journal papers only. Any formats that can be expected to only mention or superficially describe design spaces, such as proposals, panels, workshops, or doctoral consortium papers, were excluded. After a metadata-screening for relevance and removing duplicates, the number was reduced to 30 publications. A manual screening in the full text of the publications with the goal of identifying the most directly related design spaces resulted in seven final publications (Kern and Schmidt, 2009; Colley et al., 2017; Mahadevan et al., 2018; Wiegand et al., 2019; Colley and Rukzio, 2020; Graf et al., 2020; Wang and song, 2022). We used the design spaces described within them as inspirations for initial dimensions and categories. We then enriched them with features specific to capture automation as well as the human-in-the-loop characteristics to arrive at the first draft of the contextual design space.

We then evaluated this draft through a series of in-depth expert interviews with three AMHV domain experts. These experts were selected for their experience and expertise in material handling vehicles ranging from technical competence such as automation aspects to process competence that demonstrates the interrelation of various stakeholders. Experts had on average 4 years of experience working with material handling vehicles in the areas of logistics or mobility. Each interview lasted approximately 2 h, excluding preparation time. Before the interview, each interviewee was instructed to prepare a use case of their choice from within their application domain. They were free to do so in any possible way, as long as they would be able to fully describe the case and all relevant actors during the interview. The interview itself then consisted of three parts: an introduction, the design space population, and then a final feedback and comments session.

During the introduction, the interviewee was informed about the purpose of the interview as well as its duration and agenda and was then introduced to the design space, its overall purpose, as well as all dimensions and categories. They were then explicitly asked to raise questions regarding anything that was not clear before moving on to the next part. The introduction lasted 10–15 min. Then, the interviewee was asked to populate the design space with the use case they had prepared. We conducted a semi-structured interview, where the interviewer asked several predetermined thematic questions based on each part of the design space “E.g., How many individuals are involved in the overall process and which roles do they have?”. As the interviewee answered, the interviewer completed the dimensions of the design space in an Excel Sheet. This step took approximately 60–70 min. In the final phase of the interview, the interviewee was asked to reflect on the completed design space and comment on any aspects of the design space that had not been situated in the use case at all or only incompletely. They were also asked to highlight incomplete or inappropriately named labels, category errors, or any other issues that came to their mind.

On the basis of the interview results, we created an iterated version of the design space. This version was then validated in a second round of interviews with seven human-machine interaction experts. We selected our sample respondents by identifying the target population as experienced HCI designers and researchers with at least 4 years of professional experience in the field of automated vehicles. While selecting more experienced individuals might exclude the viewpoints of early-career HCI practitioners, our goal was to provide a comprehensive design space by highlighting the practical and industry-oriented insights that are crucial for the implementation of automated material-handling vehicles. These interviews were shorter, with a duration of 30–40 min each, and the interviewees were no longer asked to prepare a use case description beforehand, as this round of interviews primarily emphasized the design perspective. Instead, the interviews consisted of a very short introduction (5 min), after which the interviewer reviewed the design space together with the interviewee, asking for each dimension and its categories regarding relevance, comprehensibility, cohesion, and completeness. This part took 20–30 min. At the end, the interviewee was asked to provide a final valuation of the design space’s appropriateness as well as sum up the definitive needs for improvement, if any. The results from this second round of interviews were collected and then integrated into the final version of the contextual design space (see Table 1 for an overview of the main implications from the subsequent phases of the development of the design space).

**TABLE 1:** Overview of the implications from the literature review and the two rounds of expert interviews.

Space	Sub-space	Dimension	Main implications from the evidence collected during the design space creation process		
			Literature review	1st interview round	2nd interview round
			Previous work adopted for first draft	Resulting revisions of design space	Evidence for the finalization of the design space
**Context Space**	Purpose/task	General	Machado et al. (Machado et al., 2021a): Main Driving and handling (“manipulation”) as main categories	Refined (3rd category of coordination added)	Confirmed
		Task abstraction	-	Refined (task types and steps)	Consolidated (Michon’s model implemented, based on expert feedback (Michon, 1979))
		Degree of Freedom	-	Refined (introduced different types of DoF: actual and translated)	Consolidated (simplified DoF options)
		Duration	Wiegand et al. (Wiegand et al., 2019) introduced duration “travel time”	Introduced	Confirmed
	Automation Setting	General	-	Refined (levels of automation)	Confirmed
		Level of Automation	SAEJ3016 as widely accepted taxonomy for automated driving (Taxonomy, 2021), Machado expand this for the LoA of material handling vehicles (Machado et al., 2021a)	Refined (summarizing SAE automation levels 1/2 and 3/4)	Confirmed
		Human operator location	-	Refined (Added option “no human operator location”)	Consolidated (simplified the categories)
	Situation	General	ISO 9241-210 taxonomy for social and physical context (for Standardization, 2010); Colley et al. ‘s design space contains physical and social context variables (Colley and Rukzio, 2020)	Confirmed	Confirmed
		Social	Different user/operator roles adopted from (Graf et al., 2020; Colley and Rukzio, 2020)	Refined (added user role “maintenance”)	Consolidated (final grouping of user roles)
		Physical	Physical context aspects are broken as categories in ISO 9241-210 (for Standardization, 2010), and related to automated driving in Colley et al. (Colley and Rukzio, 2020). Soil categories were taken from (Deatherage et al., 2004). Other environmental factors, such as temperature and light intensity, were taken from (Yamazaki et al., 1998)	Refined (introduced “Dynamicity” dimension, added “sunlight” and “storm”)	Refined (Added dimension “Loading dock type”), consolidated
**Interaction Space**	Human-Automation Interaction	Scope elements Input/Output	Multiple design spaces with a differentiation of input and output (Nigay and Coutaz, 1993; Frohlich, 1992; Kern and Schmidt, 2009; Graf et al., 2020; Wang and song, 2022)	Confirmed	Confirmed
		User role profiles	-	Refined (added user role “maintenance”)	Consolidation (consistency with user role in social dimension)
		Communication type	Communication messages of automated vehicles to road users proposed by Colley et al. (Colley and Rukzio, 2020)	Confirmed	Confirmed
		Modality	Design spaces with modality as a key dimension (Detjen et al., 2021; Colley et al., 2022; Graf et al., 2020; Ahmad et al., 2018)	Confirmed	Refined (added variation “biometrics” for input modality)
		Device Type	Graf et al. propose partly propose device types, as part of their interaction space (Graf et al., 2020)	Refined (added variation “pedal”)	Confirmed
		Locus	(Detjen et al., 2021; Colley et al., 2022)	Confirmed	Confirmed
		Degree of Freedom	-	Introduced	Confirmed

## 4 Design space

In this section, we describe the design space that resulted from the iterative process described in the previous section. The design space consists of two main parts or “spaces”: *Context Space*, and *Interaction Space*.

The two spaces complement each other and also serve to reduce the complexity of any given context that is captured via the design space. The Context *Space* serves to capture all factors pertaining to the material handling context, including surface and weather constraints, machines and their automation levels, user roles and task types, *etc.* It is to be defined once for any given scenario or use case.

The *Interaction Space*, on the other hand, defines any *interaction point* within the context. An *interaction point* is any (physical) instance where a machine or human interacts. E.g., a simple context with two machines, each with one set of direct controls each as well as a fleet management workstation would result in three *interaction points* overall. The *Interaction Space* then defines in- and output for each of these points but maps back to the *Context Space* to the previously defined task types, user roles, automation setting, *etc.*

By doing so, the overall design space can efficiently capture complex human-in-the-loop scenarios with many different machine types, several control interfaces, different automation levels and intervention capabilities, without increasing exponentially. In the following, we describe the sub-space (e.g., automation setting), dimensions (e.g., driving), categories (e.g., level of autonomy), and their characteristics (e.g., semi-automated) for each space in detail.

### 4.1 Purpose/task

**FIGURE 1:**
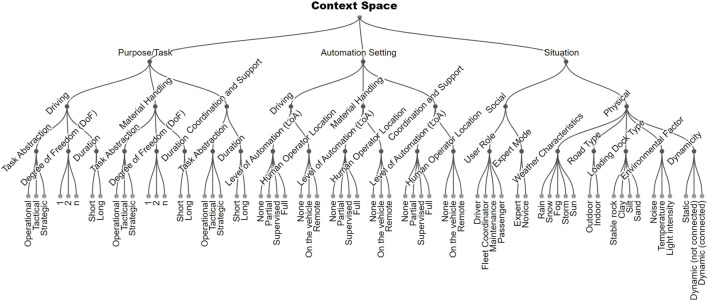
Human-Automation Context Space for AMHV (see description in sections 4.1–4.3).

Although there are two primary purposes that we cover for automated material handling vehicles, that is, driving and handling, we subdivided the purpose subspace into three scope elements: driving, handling, and support tasks (see Figure 1, left side). Driving tasks are those related to maneuvering the vehicle. Handling tasks are about handling the material such as loading/unloading the cargo. Lastly, coordination and support tasks are non-related driving or handling tasks such as management of fleet scheduling, vehicle allocation, and maintenance.

#### 4.1.1 Task abstraction

As Table 1 indicates, the scope or abstraction of a task has emerged as a relevant dimension from our first interview round, but notably, it has so far not been proposed by previous design spaces summarised in section 2. We identified three abstraction levels of driving and handling tasks, based on Michon’s model (Michon, 1979). Strategic tasks are those that plan the goal of the action such as navigation. Tactical tasks are those that facilitate the accomplishment of the task, for instance, detecting an obstacle. Operational tasks are those activities that aim to maintain and sustain a system such as loading the lattice box.

#### 4.1.2 Degree of freedom

A factor of primary relevance was found to be the physical direction in terms of movement. In order to capture this for the design space, the degree of freedom has been incorporated as a dimension of the design space. Notably, this aspect so far has not been presented as part of previous related design spaces mentioned in section 2. The most generic way to specify the target direction along their trajectories is to specify degrees of freedom, separately for the driving, handling, and support tasks (e.g., for directing vehicle charging or maintenance personnel). It has to be noted that the technical movements to be done by the handling components are typically highly complex and fine-grained (Hamid et al., 2016; Martin and Irani, 2021), thus in principle entailing many degrees of freedom. However, from the perspective of task and purpose specification, the actual defined movement targets can be specified with significantly fewer degrees of freedom, essentially reducing it towards three independent directions (x,y,z) in space.

#### 4.1.3 Duration

This dimension distinguishes between short or long duration of a task [adapted from Wiegand et al. (2019)]. According to the duration of task performance, additional interaction with the vehicle would be required. For instance, a long duration may require charging the vehicle.

### 4.2 Automation setting

For the characterization of the automation setting targeted for a certain AMHV use case, we again analyze the vehicle’s driving and handling, as well as the coordination and support activities.

#### 4.2.1 Level of automation

For categorizing automation levels for driving, handling, and coordination, we took reference to the SAE automation levels (Taxonomy, 2021) [similarly to Machado et al. (2021a)], in a condensed form.

• No automation: human operator is in direct control and performs the tasks manually (equivalent to L0 SAE J016 level).
• Partial automation: operator in direct control, but supported through partial automation (SAE L1+L2).
• Supervised automation: system is operated in an automated way, but operator should be available to intervene (SAE L3+L4).
• Full automation: system is running autonomously with interventions only in case of system errors (SAE L5).

This taxonomy is similar to the level of automation (LOA) of decision and action selection (Sheridan et al., 1978), e.g., no automation is equivalent to LOA scale 1, partial automation to LOA 2-4, supervised automation to LOA 5-9 and full automation to LOA 10.

#### 4.2.2 Human operator location

The location of operators to material handling vehicles can be either on the vehicle or distant from the vehicle, which results in different design requirements. Also in case of coordinating or supporting actions, it makes a significant difference whether the scheduling or charging is done with the vehicle in sight. This dimension emerged during the first round of interviews and was refined in the second round.

### 4.3 Situation

The situation in which the operation is undertaken will entail significant constraints on the design options for AMHV interfaces. Referring to context models from HCI and pervasive computing (ISO, 2006; for Standardization, 2010; Schmidt et al., 1999; Bradley and Dunlop, 2005; Dey, 2018), as well as to previous design spaces that had already adopted contextual dimensions (Colley et al., 2017; Wiegand et al., 2019; Colley and Rukzio, 2020; Graf et al., 2020), we include the following main elements for the situation sub-space: social and physical context (see Figure 1, right side). The first two dimensions - user roles and expertise - are related to the social context and the other five are about the physical context (weather characteristics, road type, loading dock type, environmental factor, and dynamicity).

#### 4.3.1 User role

A user is any person who is actively or passively engaged with the AMHV. The most common roles of these persons who need to be supported by AMHV user interfaces are direct control of a vehicle (driver), monitoring and coordination of (fleets of) vehicles, regular technical support, and interventions in situations of malfunction (maintenance), and passive use of a vehicle (passenger). This dimension has also been part of other design spaces (e.g., Colley and Rukzio, 2020), and the categories have been specified by means of the first round of interviews.

#### 4.3.2 Expert mode

Depending on the user interacting with the AMHV, information may embrace different degrees of detail. While the criteria in assessing expert models of operators can vary depending on the industry, equipment, and specific task, here we define expertise as the extent of specialized knowledge and skills in operating a material handling vehicle (Hetmański, 2018). This could be based on a history of successful completion of similar tasks or relevant certification or training in material handling operations. In order to capture this important difference, we identify two modes, i.e., expert and novice [adapted from Graf et al. (2020)]. For instance, a novice operator who requires the supervision of a superior is considered a novice, while an experienced operator is an expert.

#### 4.3.3 Weather characteristics

Weather as a relevant contextual dimension has been proposed for external HMIs of automated vehicles (Colley and Rukzio, 2020). We identified different characteristics such as rain, snow, and fog [adapted from Colley and Rukzio (2020)] that affect the sensor functionality. Furthermore, we added two weather characteristics that are particularly important for handling activities that emerged from the interviews: storm and sunlight.

#### 4.3.4 Road type

AMHV operation strongly depends on the road type, especially whether activities are being performed indoors or outdoors. In this regard, an indoor road can be part of a warehouse, whereas an outdoor road is outside, for instance at a construction site.

#### 4.3.5 Loading dock type

This dimension is discussed by reviewed publications. Previous work lacks a description of the soil at the loading dock (i.e., pick up or drop-off points), therefore we add a new category for specification of soil type, since loading or unloading the material can take place in locations outside of a warehouse. According to a taxonomy by the OSHA (Occupational Safety and Health Administration, Department of Labor), we identify four characteristics such as solid rock, clay, silt, and sand (Deatherage et al., 2004).

#### 4.3.6 Environmental factor

Similar to Colley and Rukzio (2020), we incorporated this category for environmental aspects that can affect the vehicle’s performance in terms of energy efficiency, component reliability, and communication interference. Two characteristics of temperature and light intensity were added to noise (Yamazaki et al., 1998), due to the relevance of this category in material handling.

#### 4.3.7 Dynamicity

This dimension is introduced during the first round of interviews. We added this category to distinguish static and dynamic environments. It influences the design of communication protocols and methods of data gathering. As AMHVs are expected to communicate with other vehicles or with infrastructure via wireless technologies, we further categorize the dynamic environment into non-connected and connected.

### 4.4 Human-automation interaction

**FIGURE 2:**
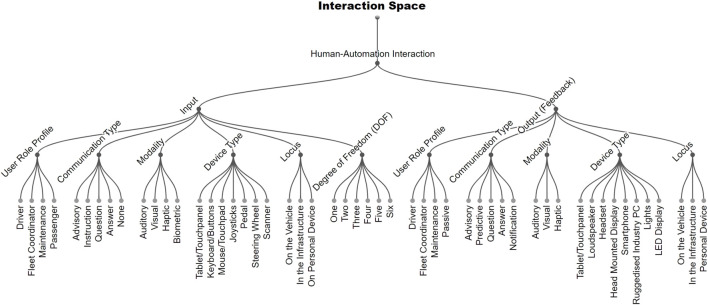
Human-Automation Interaction Space for AMHV (see description in section 4.4).

As Figure 2 shows, we propose the following dimensions to describe human automation interaction with AMHV. For both input and output, the following dimensions are proposed.

#### 4.4.1 User role profile

This dimension reflects any active (e.g., driver) or passive user (e.g., passenger) who engages in an interaction with AMHV. The most common users are the driver, who is in charge of operating the vehicle. A truck driver could also communicate with the operator, e.g., by requesting to park the AMHV at a specific destination in relation to the truck. Other users, e.g., fleet coordinator and maintenance, to some degree might interact with the AMHV. Also, a system such as a fleet management system can be considered as an active user, in case AMHV is connected to infrastructure or other machinery. Furthermore, passive users can also be considered as interacting partners. Passengers, for instance, might be informed about the activity that the AMHV is about to undertake (e.g., parking).

#### 4.4.2 Communication type

The communication type contains the elements advisory, instruction, question, answer, notification, and prediction. These characteristics are adapted from Colley and Rukzio (2020). Advisory and instruction are both guiding behaviors, however, instruction has a relatively top-down approach. Question is demanding information, while answer is providing information. Predictive is a special type of answer when the provided information contains an extent of probability. Notification is giving notice for instance about the intent of the AMHV or possible failure in executing an action such as a warning.

#### 4.4.3 Modality

This dimension is discussed by reviewing design spaces and refined during the second round of interviews. Based on previous works [e.g., (Detjen et al., 2021; Colley et al., 2022)], we identified three main interaction modalities, i.e., auditory, visual, and haptic. Auditory inputs are, for instance, speech control and non-speech sounds. Alarm or warning sounds are examples of auditory feedback. Visual inputs such as laser point or gesture are efficient for a simple command (such as selecting an option from a given alternative) (Ahmad et al., 2018). Anything displayed on the monitor or the color of vehicle lights are examples of visual feedback. Haptic controls such as pedals and the steering wheel are fixed, installed at a particular spot. Vibrations are a common example of haptic feedback (Detjen et al., 2021). Furthermore, we also include the biometric as an additional input modality for determining physical and behavioral characteristics, e.g., mental fatigue and stress of the operator (Graf et al., 2020).

#### 4.4.4 Device type

Depending on the modality of interaction, different devices can be used. For input modality, we identified tablet, keyboard, mouse, joysticks, pedal, wheel, and scanner. For the feedback modality, tablet, loudspeaker, headset, head-mounted display, smartphone, ruggedized industry PC, lights, and LED display can be listed.

#### 4.4.5 Locus

Due to the different physical positions that team members can have in relation to the vehicle, depending on the role (confer the different roles specified in the context space) and operator location (e.g., remote or on the vehicle), user interfaces may be located at different locations. Based on Mahadevan et al.’s design exploration of external communication for automated vehicles (Mahadevan et al., 2018), the locus of the interaction device can be on the vehicle, in the infrastructure, or on a user’s personal device.

#### 4.4.6 Degree of freedom

For system inputs, the degree of freedom that an interaction device offers or requires is regarded as the number of defined modes in which users can move the device to specify the command input (Albertson and Womack, 1968). For instance, the rotary knob has only one degree of freedom but a traditional mouse has two degrees of freedom. The degree of freedom has so far not been proposed as a dimension in related design spaces introduced above.

## 5 Illustrating the design space

The contextual design space introduced previously is intended to support the design of concrete instances within a flow of activities. In the case of the example of a *forestry use case* where a flatbed logging truck equipped with a z-crane is driven to a log pile in the forest and the driver uses the automated crane to load tree trunks onto the flatbed, the context is first to be specified. Table 2 lists the context parameters, highlighted in red, that apply to this use case. While driving is an operational task that occurs with three degrees of freedom and takes a long time to complete, the material handling device (i.e., the z-crane) is mechanically equipped with 6 degrees of freedom. Loading the truck with this type of handler is considered an operational task, but it takes a short completion time. Coordination and support are only necessary in the event of a fault (i.e., a defect) and can therefore be classified as an operational (manual) factor with a relatively short time impact (i.e., repair on-site). In the selected use case, the approach to automation of driving is still manual, with the driver performing the task. However, the material handling is carried out autonomously under the supervision of the user at the vehicle, while other tasks (e.g., maintenance, repairs on site) require the human to be in control of the handler or vehicle (no automation). Since in this case automation is only applicable to the handling level, the user still needs to have expert knowledge of the situation. Furthermore, the physical conditions, like weather conditions (i.e., rain or sunshine), loading dock type (i.e., silt), and environmental disturbance factors (i.e., light intensity) as well as a static dynamicity of the situation, in which the automated material handler is only communicating with the user directly, are prominent properties within the shown contextual design space.

**TABLE 2:** Context parameters of example forestry use case.

Space	Sub-space	Scope element	Dimension	Variation										
**Context Space**	Purpose/task	Driving	Task abstraction	Operational					Tactical			Strategic		
			Degree of Freedom (DoF)	1	2		3	4	5	6	7	8	9	10
			Duration	Short				Long						
		Material Handling	Task abstraction	Operational				Tactical				Strategic		
			Degree of Freedom (DoF)	1	2		3	4	5	6	7	8	9	10
			Duration	Short						Long				
		Coordination and Support	Task abstraction	Operational					Tactical			Strategic		
			Duration	Short		Long								
	Automation Setting	Driving	Level of Automation (LoA)	None				Partial		Supervised			Full	
			Human operator location	None				On the vehicle					Remote	
		Material Handling	Level of Automation (LoA)	None				Partial		Supervised			Full	
			Human operator location	None				On the vehicle					Remote	
		Coordination and Support	Level of Automation (LoA)	None				Partial		Supervised			Full	
			Human operator location	None				On the vehicle					Remote	
	Situation	Social	User roles	Driverr				Fleet coordinato		Maintenance			Passenger	
			Expertise	Expert	Novice									
		Physical	Weather characteristics	Rain			Snow	Fog		Storm			Sun	
			Road type	Outdoor					Indoor					
			Loading dock type	Stable rock			Clay	Silt			Sand			
			Environmental factor	Noise				Temperature			Light intensity			
			Dynamicity	Static				Dynamic (not connected)			Dynamic (connected)			

As shown in Table 3, on another example of a logistics use case, where lattice boxes are to be picked up by an automated forklift in a production area and then to be transported to and parked at a drop-off spot, the context can be specified following the same scheme. In this case, the operational task of driving an AMHV is relatively short in duration, but it has a medium-range degree of freedom (DOF) due to the technical and functional range of motion of the vehicle. Similar parameters also apply to the material handling and coordination tasks, although their task abstraction is classified as strategic (i.e., preventive maintenance and allocation of vehicles). A further specialty of this contextual instance is that the technical material handling scope only reflects a small range in the degree of freedom parameter field, due to the technical conditions of the material handling device (i.e., forklift). For the chosen context instance, the AMHV is assumed to have highly automated driving behavior without a human in the loop, while in the material handling task, a human is assumed to act as an external (remote) supervisor for any necessary checks and safety measures.

**TABLE 3:** Context parameters of example logistics use case.

Space	Sub-space	Scope element	Dimension	Variation										
**Context Space**	Purpose/task	Driving	Task abstraction	Operational					Tactical			Strategic		
			Degree of Freedom (DoF)	1	2		3	4	5	6	7	8	9	10
			Duration	Short				Long						
		Material Handling	Task abstraction	Operational				Tactical				Strategic		
			Degree of Freedom (DoF)	1	2		3	4	5	6	7	8	9	10
			Duration	Short						Long				
		Coordination and Support	Task abstraction	Operational					Tactical			Strategic		
			Duration	Short		Long								
	Automation Setting	Driving	Level of Automation (LoA)	None				Partial		Supervised			Full	
			Human operator location	None				On the vehicle					Remote	
		Material Handling	Level of Automation (LoA)	None				Partial		Supervised			Full	
			Human operator location	None				On the vehicle					Remote	
		Coordination and Support	Level of Automation (LoA)	None				Partial		Supervised			Full	
			Human operator location	None				On the vehicle					Remote	
	Situation	Social	User roles	Driverr				Fleet coordinato		Maintenance			Passenger	
			Expertise	Expert	Novice									
		Physical	Weather characteristics	Rain			Snow	Fog		Storm			Sun	
			Road type	Outdoor					Indoor					
			Loading dock type	Stable rock			Clay	Silt			Sand			
			Environmental factor	Noise				Temperature			Light intensity			
			Dynamicity	Static				Dynamic (not connected)			Dynamic (connected)			

In terms of the situational context in this specific logistics use case, the role of an expert user is to monitor the system, communicating over a wireless network. The situational context is furthermore characterized as dynamic, due to the changing locations of goods and other vehicles, but not as connected, as here machines are not communicating with each other. As regards the environment, the forklift operates in an indoor environment, on a stable surface such as tar or concrete. Thus, in this case, external weather characteristics are not prominent, but sunlight (shining through warehouse windows) may still be a factor of relevance, also expressed by the environmental factor of light intensity. As can be seen, the provided categories of our design space are neither exclusive nor independent. For instance, the road type for the discussed use case is both indoor and outdoor, as the boxes are handled inside a warehouse but sometimes the drop-off point is outside the warehouse building.

Based on the specified context, the possible options for human-automation interaction can be specified. Table 4 shows a task flow matrix on the example of a logistics use case. This matrix has been adapted from (Prati et al., 2021) and is completed as a result of a use case interview. As shown in Table 4, first the temporal sequence of the tasks to be performed and the actors of the actions and tasks (e.g., driver or AMHV) are defined. In our example, this process starts with the worker bringing the lattice box to the pick-up point and ends with an AMHV moving back to the parking area. In the next step, for each interaction between the user and an AMHV, the modality, device, its locus, and degree of freedom are clarified. Based on this information, designers can be supported in the exploration and allocation of a proper interplay of human actions and automated system behaviors.

**TABLE 4:** Task flow matrix of example logistics use case, used for setting the parameters for dimensions of the interaction space.

Task	User/Actor	Communication type		Modality	Device	Locus	DoF	Applicable standards
		Input	Output					
Bringing the lattice box to the pick-up point	Driver	Instruction		Haptic	Scanner	in the infrastructure	Six	EN 894
Turning on at parking area	AMHV		Notification	Visual	PC (via FMS)	in the infrastructure		EN 61310-1
Moving to the pick-up point	AMHV		Notification	Auditory	Speakers	in the infrastructure		EN 61310-1, SAE J3134
Positioning in the pick-up area	AMHV		Notification	Auditory	Speakers	on the vehicle		EN 61310-1, SAE J3134
Scanning/detection of lattice box	AMHV		Question	Visual	PC (via FMS)	in the infrastructure		EN 61310-1
Checking the lattice box for loading	Driver	Answer		Haptic	Touchpad	in the infrastructure	Two	-
Loading the lattice box	AMHV		Notification	Visual/Auditory	PC/headset	in the infrastructure		EN 61310-1
Securing the lattice box	Driver	None		Haptic	Touchpad	in the infrastructure	Two	OSHA 2236
Transporting with lattice box to the drop-off point	AMHV		Notification	Auditory	Speakers	in the infrastructure		EN 61310-1, SAE J3134
Positioning in the pick-up area	AMHV		Notification	Auditory	Speakers	on the vehicle		EN 61310-1, SAE J3134
Detecting the free spot to unload	AMHV		Advisory	Visual	Lights	on the vehicle		-
Confirming the unloading spot	Driver	Instruction		Haptic	Touchpad	in the infrastructure	Two	EN 894, EN 61310-1
Unloading the lattice box	AMHV		Notification	Visual/Auditory	PC/headset	in the infrastructure		EN 61310-1
Moving back to parking area	AMHV		Notification	Visual	PC (via FMS)	in the infrastructure		EN 61310-1, SAE J3134

At the very right column of the task flow matrix in Table 4, standards are provided, which have to be considered or followed when addressing the interaction design regarding a certain task, and thus these can impose potential design constraints. The referenced standards include design requirements for heavy machinery, such as principles for visual, acoustic, and tactile signals (European Machine Directive, EN61310-1 (for Electrotechnical Standardization, 2008a)), for visual displays and control actuators (EN 894-1:1997 + A1:2008 (for Electrotechnical Standardization, 2008c)), and for indications, actuation and marking (EN61310-2 (for Electrotechnical Standardization, 2008b)). Also, standards applying for the general scope of automated driving are to be considered, most importantly the SAE J3134 for vehicle lighting towards other road users (SAE, 2019). As regards operational health standards for the material handling domain, respective standards like OSHA 2236 (Safety and Administration, 2002) also need to be taken into account in the design process.

## 6 Discussion

In the following, we discuss the in-practice application of the design space, as well as two related aspects, namely, the capturing of human-in-the-loop aspects as well as the feasibility of strict separation of the design space constituents.

### 6.1 Making use of the design space

The primary purpose and intended use of the design space is to situate any given interaction context or specific challenge within it and then identify the most suitable design options in a structured way. In doing so, one can reveal, identify, and categorize all relevant aspects (objects, actors, or parameters) that can (a) be subject to or targets of design activities, (b) influence interactions, including the success of interaction designs within the context (Dove et al., 2016). This aligns with the notion of a design space also being able to serve as a practical foundation for promising and novel, but also challenging and still insufficiently structured interface classes or application areas (Ballagas et al., 2008; Kern and Schmidt, 2009; Tönnis et al., 2009; Haeuslschmid et al., 2016; Braun et al., 2017; Wiegand et al., 2019; Colley and Rukzio, 2020).

While only recently previous design spaces have started to add contextual variables (Colley and Rukzio, 2020), the AMHV context space necessarily had to be more comprehensive, given the multitude of possible situations, automation settings, and allocated tasks. Mapping out an entire use case within a specific context can be time-consuming, which is why the space is modular and should be used as such: The *Context Space* can be used on its own to capture possible influences on any given design activity and be used as a design aid even when the interaction space is not being used. Users can already gain all relevant information regarding contextual variables, possible task types, controllability of machines, and their degree of automation, as well as elementary user characteristics.

The *Interaction Space* can be used to finely detail any given interaction situation. It is intended to be detailed for any given point of interaction, e.g.,: if there are two machines, a fleet management interface for both, and each with its own on-machine control interface, then that results in three interaction points overall. As a result, a full capturing of this space would entail specifying input and output three times, separately for each interaction point. Thereby, the interaction space is specified in accordance with both the level of interactional complexity in the specific use case as well as the design needs - if the design for a specific interaction point is out of the scope of the current activities, then that one can be omitted.

### 6.2 Capturing the human(s) in the loop

Describing the involvement of humans in automated processes in the area of material handling is especially complex, as work roles and team allocations are currently evolving (Cimini et al., 2020). One of the bigger challenges of creating the design space was thus to capture the human role within various scenarios of automation, without introducing needless complexity into the design space, as the space needs to be easily readable and graspable in order to serve its primary purpose (Halskov et al., 2021). Since the context space is defined once for a given use case and the size of the interaction space is proportional to the number of interaction points, we aimed to contain the human in the loop within the context space as much as possible, in order to keep complexity low.

In our approach, we captured the aspects relevant to the position of the human in the loop via the level of abstraction as well as the automation setting for each task (driving, handling, coordination, and support). While this does not result in detailed human-machine-interaction workflows with exact indicators as to when and where the human is involved to which capacity, it does provide a similar result once the interaction space maps back to it: Since the level of automation–and with it, the degree of human involvement—are specified in the context space, this information does not need to be repeated for every single interaction point. Even high-level task durations are already specified in the context space already. Also, the human operator location is specified for the driving, handling, and coordination and support tasks, which gives, in combination, an overall impression of the distribution of human-automation task distribution among the team. Thus, by mapping the interaction to the context space, any given interaction is specified regarding the Where, When, and How of the Human-in-the-Loop.

This approach does have two drawbacks: The temporal component is high-level and specific task durations or times when certain tasks are performed are not supported by this design space. In addition, it is not possible to specify different levels of involvement within the same interaction point and user role, which can occur in individual cases (e.g., different levels of experience between two individuals sharing the same role leading to different involvement). Especially the latter is very specific and out of the scope of a typical design space (Simon, 1975; Halskov et al., 2021). Still, both are relevant to finely specify the role and position of the Human-in-the-Loop, thereby also suggesting a limit as to how far this can be specified within a design space alone.

### 6.3 Managing definitions and delimitations

A design space, at least in its classical understanding (Shaw, 2012), implies that its dimensions are independent and that parameters along these dimensions should be discrete. However, in system types such as mechanical material handling vehicles, delicate interdependencies need to be considered (Halskov et al., 2021). We encountered a fundamental example of this during the creation of the AMHV design space, as we were separating driving from handling as the two elementary task categories. While both involve movement to some extent, focus and challenge are different: Movement is primarily a matter of (two-dimensional) trajectory planning, steering maneuver execution, and dealing with different surface types. Material handling, however, involves trajectories in a three-dimensional space, with challenges more related to picking up and putting down, and also concerned with the type and quantity of material to be handled, as well as generally shorter trajectories. In addition, there is also frequently a clear physical distinction between a machine’s driving and handling means (e.g., wheels vs. crane boom).

We had separated the two categories like that in the initial draft already and the division held until the final version, with iterations mainly concerning the dimensions and their refinement. What became clear, however, was that a clear separation was sometimes more challenging in practice and the term “movement” could sometimes be misleading. Two machine types where this came up more frequently were forklifts and swap-body trucks. Forklifts do have a clear separation between fork movement and forklift steering controls. However, part of the picking-up motion is purely driving: The forklift is first, via the regular driving controls, maneuvered into position so that the fork is positioned below the stillage. Only then is the fork moved upwards. The question is then - how should the initial maneuvering be classified: as movement or material handling? Swap-body trucks face a very similar challenge. Such trucks simply dock at a loading station, where their body is then loaded automatically. A truck can dock onto any loaded body and drive out for delivery, hence the term “swap body”. The question is whether the driving into the docking station constituted a driving or a material handling task.

One possible solution to this problem could be to further define tasks specific to their purpose. This could mean that if an action is executed primarily for the purpose of handling or preparing to handle material, then it would be classified under material handling, even if it uses driving controls and maneuvers only. If, on the other hand, the primary purpose is the navigation of the entire machine from point A to point B, then it would be classified under driving. The challenge with this solution is the clear delineation in specific cases - where does the driving end and how much of the approach of, e.g., the forklift to the hall where the stillages are, is handling? Given that there is no clear intrinsic distinction, this would need to be defined at least on a machine-level separate for each machine type, perhaps even on a contextual level (storehouse types, *etc.*), which would defeat the purpose of a design space that should not impose unnecessary restrictions and enable consistency.

Instead, we decided and subsequently suggest to separate the task categories on the control level instead. If the task is executed via driving controls and entails moving the machine, it is of the driving type. If it is executed via non-driving controls and either directly involves or has the immediate purpose of handling material (including repositioning), then it is classified as the material handling type. This means that both the initial maneuvering of the forklift, as well as the entire docking operation of a swap body truck, would be classified as driving. On the interaction level especially, this renders the distinction clear, as there is no switch from driving to handling on the same set of controls. For the swap body trucks in particular, it would seem that this categorization then misses the material handling component entirely. However, the actual material bulk of the material handling challenge in these cases happens during the container loading operation, where the truck is simply not involved, and not during docking. As such, the categorization also more adequately reflects the extent of material handling involved, which is minimal to nonexistent in these cases.

## 7 Limitations

The design space was based on a foundation of existing design spaces and was iterated on the basis of expert inputs from professionals working in AMHV contexts as well as HCI. Due to the often closed nature of industrial AMHV use cases and the resulting difficulty of stakeholder access, gathering the ten experts involved was already very challenging. While in line with or even above the number of experts involved when creating a design space (Braun et al., 2017; Wiegand et al., 2019; Colley and Rukzio, 2020), it still means that the number of individuals involved was on the lower end. While the application domains we focused on (construction, agriculture, intralogistics, and manufacturing) represent a broad spectrum of material handling applications, we do expect that applications outside of the investigated domains will yield further requirements or extensions for the design space. To this end, one should keep in mind that a design space should serve as a design aid that should be adaptable along each tackled design project (Heape, 2007). A particularly promising area of further extending the AMHV design space has been proposed by (Steckhan et al., 2022), suggesting the extension of lower-level abstractions (e.g., functional driving dynamics as cues for interventions) and user satisfaction as target functions. Another limitation lies with our separation of task types between driving and material handling and delineating the two on the control level. While this solution does lead to a clearer distinguishability and reflects the involved actual material handling well, it cannot appropriately capture some corner cases, such as, e.g., using a crane boom to push oneself away, thus constituting movement rather than any type of handling operation. While such actions are typically outside of the intended scope (and unsafe as well as nonpermitted as a result), capturing non-intended use can be very valuable for accurately describing design contexts (Satchell and Dourish, 2009) and we consider this potential room for improvement.

## 8 Conclusion

In this paper, we presented a design space for AMHV. The design space is based on six existing design spaces for either automation or material handling and is the first design space that captures both aspects and enables AMHV contexts to be fully situated within. The design space consists of two sub-spaces—the Context Space and the Interaction Space. This division enables efficient definition of each interaction point in the Interaction Space by mapping back to the contextual factors (user roles, task types, level of automation, *etc.*) that are globally defined in the Context Space. The design space can be used to support targeted design efforts that in configurations are characteristic of automated material handling use cases, including extended process chains and multiple interaction chains across several machines that involve different user roles, remote vs. on-machine operation, as well as different degrees of automation and corresponding intervention or monitoring capabilities. It is the first dedicated design space specific to AMHV and shall serve to be a useful tool for future design efforts as well as provide a consistent framing for AMHV contexts going forward.

## 9 Future work

One of the main goals of this design space was to provide a tool to structure any given context in order to then situate one’s design activities within it and to identify correct devices, locus, users, *etc.* We plan to conduct prototyping-oriented research with the help of the proposed design space, specifically focusing usability and acceptability of fleet monitoring interfaces in multi-machine contexts. We use the design space to mainly capture the type and levels of automation and controllability for each machine involved, then identify and design for the user roles that require access to the fleet view, with the eventual goal of defining views with separate indicators and different levels of detail, depending on physical location and which user roles access it.

## Data Availability

The raw data supporting the conclusions of this article will be made available by the authors, without undue reservation.

## References

[B1] Ahmad B. I. Hare C. Singh H. Shabani A. Lindsay B. Skrypchuk L. (2018). “Selection facilitation schemes for predictive touch with mid-air pointing gestures in automotive displays,” in Proceedings of the 10th international conference on automotive user interfaces and interactive vehicular applications, Toronto, Canada, September 23 - 25, 2018, 21–32.

[B2] Albertson H. Womack B. (1968). Input and output degrees of freedom of linear systems. IEEE Trans. Automatic Control 13, 745. 10.1109/TAC.1968.1099053

[B3] Ballagas R. Rohs M. Sheridan J. G. Borchers J. (2008). “The design space of ubiquitous mobile input,” in Handbook of research on user interface design and evaluation for mobile technology (Pennsylvania, United States: IGI Global), 386–407.

[B4] Bauer C. Novotny A. (2017). A consolidated view of context for intelligent systems. J. Ambient Intell. Smart Environ. 9, 377–393. 10.3233/ais-170445

[B5] Beaudouin-Lafon M. Mackay W. E. (2009). “Prototyping tools and techniques,” in Human-computer interaction (Florida, United States: CRC Press), 137–160.

[B6] Benos L. Bechar A. Bochtis D. (2020). Safety and ergonomics in human-robot interactive agricultural operations. Biosyst. Eng. 200, 55–72. 10.1016/j.biosystemseng.2020.09.009

[B7] Bevan N. Carter J. Harker S. (2015). “Iso 9241-11 revised: what have we learnt about usability since 1998?,” in International conference on human-computer interaction, Bamberg, Germany, September 14-18, 2015 (Springer), 143–151.

[B8] Biskjaer M. M. Dalsgaard P. Halskov K. (2014). “A constraint-based understanding of design spaces,” in Proceedings of the 2014 Conference on Designing Interactive Systems (New York, NY, USA: Association for Computing Machinery), DIS ’14, Vancouver, BC, Canada, June 21-25, 2014 (New York, NY, USA: Association for Computing Machinery), 453–462. 10.1145/2598510.2598533

[B9] Bradley N. A. Dunlop M. D. (2005). Toward a multidisciplinary model of context to support context-aware computing. Human-Computer Interact. 20, 403–446. 10.1207/s15327051hci2004_2

[B10] Braun M. Broy N. Pfleging B. Alt F. (2017). “A design space for conversational in-vehicle information systems,” in Proceedings of the 19th International Conference on Human-Computer Interaction with Mobile Devices and Services, Vienna, Austria, 1–8 September, 2017.

[B11] Buxton W. (1983). Lexical and pragmatic considerations of input structures. SIGGRAPH Comput. Graph. 17, 31–37. 10.1145/988584.988586

[B12] Card S. Mackinlay J. (1997). “The structure of the information visualization design space,” in Proceedings of VIZ ’97: Visualization Conference, Information Visualization Symposium and Parallel Rendering Symposium, Phoenix, AZ, USA, 21 October 1997, 92–99. 10.1109/INFVIS.1997.636792

[B13] Card S. K. Mackinlay J. D. Robertson G. G. (1991). A morphological analysis of the design space of input devices. ACM Trans. Inf. Syst. 9, 99–122. 10.1145/123078.128726

[B14] Chen J. Y. Barnes M. J. (2014). Human–agent teaming for multirobot control: a review of human factors issues. IEEE Trans. Human-Machine Syst. 44, 13–29. 10.1109/thms.2013.2293535

[B15] Chi E. (2000). “A taxonomy of visualization techniques using the data state reference model,” in IEEE Symposium on Information Visualization 2000. INFOVIS 2000. Proceedings, Salt Lake City, Utah, 09-10 October 2000, 69–75. 10.1109/INFVIS.2000.885092

[B16] Cimini C. Lagorio A. Romero D. Cavalieri S. Stahre J. (2020). Smart logistics and the logistics operator 4.0. IFAC-PapersOnLine 53, 10615–10620. 10.1016/j.ifacol.2020.12.2818

[B17] Colley A. Häkkilä J. Pfleging B. Alt F. (2017). “A design space for external displays on cars,” in Proceedings of the 9th International Conference on Automotive User Interfaces and Interactive Vehicular Applications Adjunct, Oldenburg, Germany, September 24 - 27, 2017, 146–151.

[B18] Colley M. Mytilenaios S. Walch M. Gugenheimer J. Rukzio E. (2022). Requirements for the interaction with highly automated construction site delivery trucks. Front. Hum. Dyn. 4. 10.3389/fhumd.2022.794890

[B19] Colley M. Rukzio E. (2020). “A design space for external communication of autonomous vehicles,” in 12th International Conference on Automotive User Interfaces and Interactive Vehicular Applications, Washington, DC, USA, 21 - 22 September 2020, 212–222.

[B20] Costello B. Suarez R. (2015). Truck driver shortage analysis 2015. Arlington, VA: The American Trucking Associations.

[B21] Deatherage J. H. Furches L. K. Radcliffe M. Schriver W. R. Wagner J. P. (2004). Neglecting safety precautions may lead to trenching fatalities. Am. J. Industrial Med. 45, 522–527. 10.1002/ajim.20010 15164396

[B22] Detjen H. Faltaous S. Pfleging B. Geisler S. Schneegass S. (2021). How to increase automated vehicles’ acceptance through in-vehicle interaction design: a review. Int. J. Human–Computer Interact. 37, 308–330. 10.1080/10447318.2020.1860517

[B23] Dey A. K. (2018). “Context-aware computing,” in Ubiquitous computing fundamentals (Florida, United States: Chapman and Hall/CRC), 335–366.

[B24] Dove G. Hansen N. B. Halskov K. (2016). “An argument for design space reflection,” in Proceedings of the 9th Nordic Conference on Human-Computer Interaction (New York, NY, USA: Association for Computing Machinery), NordiCHI ’16, New York, NY, USA, October 25 - 29, 2020 (New York, NY, USA: Association for Computing Machinery). 10.1145/2971485.2971528

[B25] Efthymiou O. K. Ponis S. T. (2019). Current status of industry 4.0 in material handling automation and in-house logistics. Int. J. Industrial Manuf. Eng. 13, 1382–1386.

[B26] for Electrotechnical Standardization E. C. (2008a). En 61310-1:2008safety of machinery — indication, marking and actuation — part 1: requirements for visual, acoustic and tactile signals (iec 61310-1:2007), 26.

[B27] for Electrotechnical Standardization E. C. (2008b). En 61310-2:2008 safety of machinery -indication, marking and actuation - part 2: requirements for marking (iec 61310-2:2007), 26.

[B28] for Electrotechnical Standardization E. C. (2008c). En 894-1:1997+a1:2008 safety of machinery - ergonomic requirements for the design of displays and control actuators, 26.

[B29] for Standardization I. O. (2010). *Ergonomics of human-system interaction: Part 210: human-centred Design for interactive systems* (ISO).

[B30] Fottner J. Clauer D. Hormes F. Freitag M. Beinke T. Overmeyer L. (2021). Autonomous systems in intralogistics–state of the art and future research challenges. J. Logist. Res.

[B31] Frank M. (2019). A step towards the design of collaborative autonomous machines: a study on construction and mining equipment (Karlskrona, Sweden: Blekinge Tekniska Högskola). Ph.D. thesis.

[B32] Frohlich D. M. (1992). “The design space of interfaces,” in Multimedia (Cham: Springer), 53–69.

[B33] Gil M. Albert M. Fons J. Pelechano V. (2019). Designing human-in-the-loop autonomous cyber-physical systems. Int. J. human-computer Stud. 130, 21–39. 10.1016/j.ijhcs.2019.04.006

[B34] Graf G. Palleis H. Hussmann H. (2020). A design space for advanced visual interfaces for teleoperated autonomous vehicles. In Proceedings of the International Conference on Advanced Visual Interfaces. 1–3.

[B35] Gupta V. Mongrain D. Tosato P. Varanasi S. (2022). A changing material-handling market: how to ensure continuous success. Seattle, Washington: McKinsey.

[B36] Ha Q. Yen L. Balaguer C. (2018). “Earthmoving construction automation with military applications: past, present and future,” in ISARC 2018-35th International Symposium on Automation and Robotics in Construction and International AEC/FM Hackathon: The Future of Building Things.

[B37] Haeuslschmid R. Pfleging B. Alt F. (2016). “A design space to support the development of windshield applications for the car,” in Proceedings of the 2016 CHI Conference on Human Factors in Computing Systems, 5076–5091.

[B38] Halskov K. Dove G. Fischel A. (2021). Constructing a design space from a collection of design examples. She Ji J. Des. Econ. Innovation 7, 462–484. 10.1016/j.sheji.2021.07.001

[B39] Hamid M. Jamil M. Gilani S. O. Ikramullah S. Khan M. N. Malik M. H. (2016). Jib system control of industrial robotic three degree of freedom crane using a hybrid controller. system 500, 7. 10.17485/ijst/2016/v9i21/94813

[B40] Heape C. (2007). The design space: the design process as the construction, exploration and expansion of a conceptual space.

[B41] Heath T. (2018). Autonomous industrial machines and the effect of autonomy on machine safety. Master’s thesis.

[B42] Heikkilä R. Makkonen T. Niskanen I. Immonen M. Hiltunen M. Kolli T. (2019). “Development of an earthmoving machinery autonomous excavator development platform,” in ISARC. Proceedings of the International Symposium on Automation and Robotics in Construction (IAARC Publications), vol. 36, 1005–1010.

[B43] Hetmański M. (2018). Expert knowledge: its structure, functions and limits. Stud. Humana 7, 11–20. 10.2478/sh-2018-0014

[B44] Hoffmann E. R. Chan A. H. S. (2018). Review of compatibility and selection of multiple lever controls used in heavy machinery. Int. J. Industrial Ergonomics 65, 93–102. 10.1016/j.ergon.2017.07.007

[B45] Hopkins D. Schwanen T. (2021). Talking about automated vehicles: what do levels of automation do? Technol. Soc. 64, 101488. 10.1016/j.techsoc.2020.101488

[B46] ISO (2006). Iso 20282-1: 2006 ease of operation of everyday products—part 1: design requirements for context of use and user characteristics.

[B47] Kern D. Schmidt A. (2009). “Design space for driver-based automotive user interfaces,” in Proceedings of the 1st International Conference on Automotive User Interfaces and Interactive Vehicular Applications, 3–10.

[B48] Krug A. Seidel P. Knoblinger T. (2019). Autonomous machines in the fast lane? Arthur D Little.

[B49] Lee J. S. Ham Y. Park H. Kim J. (2022). Challenges, tasks, and opportunities in teleoperation of excavator toward human-in-the-loop construction automation. Automation Constr. 135, 104119. 10.1016/j.autcon.2021.104119

[B50] Li M. Katrahmani A. Kamaraj A. V. Lee J. D. (2020). “Defining a design space of the auto-mobile office: a computational abstraction hierarchy analysis,” in Proceedings of the Human Factors and Ergonomics Society Annual Meeting (SAGE Publications Sage CA: Los Angeles, CA), vol. 64, 293–297.

[B51] Li M. P. Sankaran P. Kuhl M. E. Ganguly A. Kwasinski A. Ptucha R. (2018). “Simulation analysis of a deep reinforcement learning approach for task selection by autonomous material handling vehicles,” in Proceedings of the 2018 Winter Simulation Conference (IEEE Press), WSC ’18, 1073–1083.

[B52] Machado T. Ahonen A. Ghabcheloo R. (2021a). “Towards a standard taxonomy for levels of automation in heavy-duty mobile machinery,” in Fluid power systems technology (New York City: American Society of Mechanical Engineers). vol. 85239, V001T01A055.

[B53] Machado T. Fassbender D. Taheri A. Eriksson D. Gupta H. Molaei A. (2021b). “Autonomous heavy-duty mobile machinery: a multidisciplinary collaborative challenge,” in 2021 IEEE International Conference on Technology and Entrepreneurship (ICTE) (IEEE), 1–8.

[B54] MacLean A. Young R. M. Bellotti V. M. Moran T. P. (2020). “Questions, options, and criteria: elements of design space analysis,” in Design rationale (Florida, United States: CRC Press), 53–105.

[B55] Mahadevan K. Somanath S. Sharlin E. (2018). “Communicating awareness and intent in autonomous vehicle-pedestrian interaction,” in Proceedings of the 2018 CHI Conference on Human Factors in Computing Systems. 1–12.

[B56] Martin I. A. Irani R. A. (2021). Dynamic modeling and self-tuning anti-sway control of a seven degree of freedom shipboard knuckle boom crane. Mech. Syst. Signal Process. 153, 107441. 10.1016/j.ymssp.2020.107441

[B57] Merat N. Seppelt B. Louw T. Engström J. Lee J. D. Johansson E. (2019). The “out-of-the-loop” concept in automated driving: proposed definition, measures and implications. Cognition, Technol. Work 21, 87–98. 10.1007/s10111-018-0525-8

[B58] Michon J. A. (1979). Dealing with danger. Tech. Rep., verkeerskundig studiecentrum, rijksuniversiteit groningen haren, The Netherlands.

[B59] Müller J. Alt F. Michelis D. Schmidt A. (2010). “Requirements and design space for interactive public displays,” in Proceedings of the 18th ACM International Conference on Multimedia (New York, NY, USA: Association for Computing Machinery), MM ’10, 1285–1294. 10.1145/1873951.1874203

[B60] Nigay L. Coutaz J. (1993). “A design space for multimodal systems: concurrent processing and data fusion,” in Proceedings of the INTERACT ’93 and CHI ’93 Conference on Human Factors in Computing Systems (New York, NY, USA: Association for Computing Machinery), CHI ’93, 172–178. 10.1145/169059.169143

[B61] Prati E. Peruzzini M. Pellicciari M. Raffaeli R. (2021). How to include user experience in the design of human-robot interaction. Robotics Computer-Integrated Manuf. 68, 102072. 10.1016/j.rcim.2020.102072

[B62] Rogers W. P. Kahraman M. M. Drews F. A. Powell K. Haight J. M. Wang Y. (2019). Automation in the mining industry: review of technology, systems, human factors, and political risk. Min. Metallurgy Explor. 36, 607–631. 10.1007/s42461-019-0094-2

[B63] SAE (2019). J3134_201905 automated driving system (ADS) marker lamp, 26.

[B64] Safety O. O. Administration H. (2002). Materials handling and storage, 41.

[B65] Satchell C. Dourish P. (2009). “Beyond the user: use and non-use in HCI,” in Proceedings of the 21st Annual Conference of the Australian Computer-Human Interaction Special Interest Group: Design: Open 24/7 (New York, NY, USA: Association for Computing Machinery), OZCHI ’09, 9–16. 10.1145/1738826.1738829

[B66] Schmidt A. Beigl M. Gellersen H.-W. (1999). There is more to context than location. Comput. Graph. 23, 893–901. 10.1016/s0097-8493(99)00120-x

[B67] Shaw M. (2012). The role of design spaces. IEEE Softw. 29, 46–50. Conference Name: IEEE Software. 10.1109/MS.2011.121

[B68] Sheridan T. B. Verplank W. L. Brooks T. (1978). “Human/computer control of undersea teleoperators,” in NASA. Ames Res. Center The 14th Ann. Conf. on Manual Control.

[B69] Simon H. A. (1975). Style in design. Spatial synthesis computer-aided Build. Des. 9, 287–309.

[B70] Steckhan L. Spiessl W. Quetschlich N. Bengler K. (2022). “Beyond sae j3016: new design spaces for human-centered driving automation,” in International Conference on Human-Computer Interaction (Springer), 416–434.

[B71] Taxonomy S. (2021). Definitions for terms related to driving automation systems for on-road motor vehicles-. publication j3016_202104. Society of Automotive Engineers.

[B72] Tönnis M. Klinker G. Plavšic M. (2009). Survey and classification of head-up display presentation principles. Proc. Int. Ergonomics Assoc. (IEA).

[B73] Vagia M. Transeth A. A. Fjerdingen S. A. (2016). A literature review on the levels of automation during the years. what are the different taxonomies that have been proposed? Appl. Ergon. 53, 190–202. 10.1016/j.apergo.2015.09.013 26467193

[B74] Wang W. song F. Zhu T. (2022). Same: the design space for seamless automotive multimodal experience. IT Prof. 24, 35–42. 10.1109/mitp.2022.3172943

[B75] Wang W. Song F. Zhu T. (2022). Same: the design space for seamless automotive multimodal experience. IT Prof. 24, 35–42. 10.1109/MITP.2022.3172943

[B76] Wankhede V. A. Vinodh S. (2021). State of the art review on industry 4.0 in manufacturing with the focus on automotive sector. Int. J. Lean Six Sigma. 10.1108/IJLSS-05-2021-0101

[B77] Wiegand G. Mai C. Holländer K. Hussmann H. (2019). “Incarar: a design space towards 3d augmented reality applications in vehicles,” in Proceedings of the 11th international conference on automotive user interfaces and interactive vehicular applications. 1–13.

[B78] Yamazaki K. Nomoto S. Yokota Y. Murai T. (1998). The effects of temperature, light, and sound on perceived work environment. ASHRAE Trans. 104, 711.

